# Parathyroid Carcinoma: The Importance of High Clinical Suspicion for a Correct Management

**DOI:** 10.1155/2012/649148

**Published:** 2012-09-19

**Authors:** Gabriele Ricci, Marco Assenza, Marco Barreca, Gianluca Liotta, Livio Paganelli, Angelo Serao, Giovanni Tufodandria, Pierluigi Marini

**Affiliations:** ^1^Division of Endocrine and Bariatric Surgery, San Camillo-Forlanini Hospital, Piazza Carlo Forlanini 1, 00151 Rome, Italy; ^2^Division of Emergency surgery and Trauma, Sapienza University of Rome Umberto I General Hospital, Viale del Policlinico 155, 00161 Rome, Italy

## Abstract

*Background*. Parathyroid carcinoma is an infrequent clinical entity whose diagnosis is very challenge. Indeed a pre-operative or intraoperative diagnosis of parathyroid carcinoma is reported in less than half cases described in the literature. *Patients and Methods*. A systematic review of pathological reports of our secondary referral hospital was done. From 2003 to 2011 one hundred and forty-four patients were operated for hyperparathyroidism. One patient with atypical adenoma and three patients with parathyroid carcinoma were included in this paper. *Results*. An *en bloc* resection of the tumor was performed in three patients. Two of this patients with diagnosis of parathyroid carcinoma are alive with no evidence of recurrence or metastasis, respectively, 48 and 60 months after the operation; one patient with diagnosis of atypical adenoma died for other disease 16 months after the operation. In the last patient a simple parathyroidectomy was performed. After that histology revealed the diagnosis of parathyroid carcinoma the patient underwent reoperation for left hemithyroidectomy and central compartment lymph node clearance. After 30 months a lung lobectomy was done due to metastasis. *Conclusion*. Parathyroid carcinoma should be considered in the differential diagnosis of PTH-dependent hypercalcemia because optional outcomes are associated with complete resection of the tumor at the time of initial operation.

## 1. Introduction

Parathyroid carcinoma is an infrequent clinical entity accounting for 0,5% to 5% of all cases of primary hyperparathyroidism [1–4]. It represents a diagnostic and therapeutic challenge because this rare endocrine malignancy usually is not recognized preoperatively and often is not conclusively identified during the operation or at histological examination. Despite its rarity it is very important for every endocrine surgeon to be aware of the disease because the initial *en bloc* resection represents the only chance for cure [1–8].

The objective of this paper is to elucidate the importance of high clinical suspicion in operative management of parathyroid carcinoma, analyzing retrospectively the cases treated in our service.

## 2. Patients and Methods

A systematic review of pathological reports of our secondary referral hospital was done. From 2003 and 2011 one hundred and forty-four patients were operated for hyperparathyroidism at our service. Histology distribution of patients is reported in Table 1. One patient with atypical adenoma and three patients with the final diagnosis of parathyroid carcinoma were included in this review. None had any family history of hyperparathyroidism or history of neck irradiation. They had one or more absolute criterion of malignancy: pathological lymphatic or vascular invasion, capsular breach to invade adjacent neck structures, local recurrence or regional/distant metastasis. Main clinical features and surgical management are listed in Table 2.

### 2.1. Patient 1

A 46-year-old women was admitted in our department with diagnosis of primary hyperparathyroidism (PHPT). In her past medical history two episodes of nephrolithiasis were reported, respectively, 3 years and 1 year ago. Since 6 months she reported fatigue, bone and muscular pain, and anxiety. The diagnosis of PHPT was established with calcium and parathormone (PTH) levels of 14,0 mg/dL, and 898 pg/mL respectively. Ultrasound scan identified a left superior thyroid lobe mass measuring 2,7 × 1,4 × 2,5 cm. Tc-99m sestamibi scan confirmed a persistent focus of activity in the same area. During neck exploration a firm grayish mass adherent to left thyroid lobe was found. A left thyroid lobectomy with paratracheal lymphadenectomy was done. Postoperative course was complicated by severe hypocalcaemia that required hospitalization for 10 days for intravenous administration of calcium. Final pathology report was consistent with the diagnosis of parathyroid carcinoma. Histology showed the presence of dense fibrous bands with cells arranged in a trabecular pattern infiltrating into the thyroid tissue; vessel invasion was also evident. At follow up to 5 years the patient was in good clinical conditions and free of recurrence.

### 2.2. Patient 2

A 69-year-old man presented with well-known long history of osteoporosis and renal stone. Laboratory findings showed serum calcium level of 13,0 mg/dL and PTH levels of 184 pg/mL. Ultrasonography of the neck revealed a 1,5 cm extrathyroidal parenchymal lump in the left superior thyroid lobe. Tc-99m sestamibi scan confirmed a persistent focus of activity in the same area. Radio-guided minimally invasive parathyroidectomy (MIVAP) was performed; no intraoperative abnormalities were found. Histological report showed parathyroid carcinoma with foci of capsular and vascular invasion. The patient underwent reoperation three weeks later for left hemithyroidectomy and central compartment lymph node clearance. Histology demonstrated the absence of tumor in the new resected specimen; no lymph node metastasis were found. Immediately after the operation serum calcium and PTH returned to the normal range. 

30 months later rise in follow-up serum calcium and PTH levels were noted. Ultrasonography of the neck showed no abnormalities, but Tc-99m sestamibi scan detected marked increased radiotracer activity in the region of lower right lung lobe. CT scan confirmed the presence of 1 cm mass in the lower right lung lobe, then lung lobectomy was done. After operation serum calcium and PTH levels reduced but did not return to normal range (10,7 mg/dL, and 96 pg/mL resp.). Further investigations (neck ultrasonography, neck and chest CT, Tc-99m sestamibi scan and PET scan) fail to demonstrate any site of recurrence or metastasis until now. 

### 2.3. Patient 3

A 64-year-old man was referred to our hospital with a diagnosis of PHPT. His history dated back two years with nephrolithiasis complicated by renal impairment and spontaneous fracture of inferior limb. The patient complained for some weeks the presence of a lump in the left neck. Serologies showed a serum calcium level of 14,2 mg/dL and a preoperative intact PTH of 620 pg/mL. Ultrasound identified an hypoechoic nodule 3,6 × 2 × 2,7 cm behind the left lobe of the thyroid. Tc-99m sestamibi scan resulted negative; however a Tl-201-Tc-99m scan located a marked increased radiotracer activity in the region of the inferior left lobe of the thyroid. At surgery a large-firm, hard, and lobulated mass adherent to the thyroid and to the strap muscle was found. *En bloc* resection of the mass, the strap muscle. and the ipsilateral thyroid lobe was performed. Postoperative course was complicated by hypocalcaemia. Histology revealed the diagnosis of parathyroid carcinoma; pathology report described a gray-white hard tumor mass, 3,8 cm in maximum diameter, with a thick fibrous capsule surrounded it. Microscopic examination noted monomorphous parathyroid cells arranged in a trabecular solid architecture, with numerous mitotic figures, showing invasion beyond its capsule into the adjacent strap muscle and fatty tissue. Micronodular goiter was also described in the thyroid lobe. At followup to 4 years the patient was in good clinical conditions and free of recurrence.

### 2.4. Patient 4

A 54-year-old man with symptoms of weakness, lethargy, bone pain secondary to osteoporosis and palpable neck mass was admitted in our department. PHPT diagnosis was established based on the serum calcium and intact PTH level of 13,2 mg/dL, and 475 pg/mL respectively. Ultrasound scan and Tc-99m sestamibi scan of the neck identified a mass behind the right thyroid lobe. At surgery a gray-white fibrous capsule adherent to the base of the inferior right lobe of the thyroid was found; therefore an *en bloc* resection of the mass with the ipsilateral thyroid lobe was performed. Histology was consistent with atypical adenoma; the tumor was noted to be composed of lobules of parathyroid cells with moderate mitotic activity and thick fibrous bands. Abnormal mitotic figures and capsular invasion were apparent, but there were no clear signs of vessel or surrounding tissue invasion. At followup after 16 months the patient died for other disease.

## 3. Discussion

Parathyroid carcinoma is a rare cause of PHPT. Its rarity limits reports to small institutional series with occasional reviews of all experiences reported in the literature. The majority of patients with parathyroid carcinoma present in their fifties, and unlike parathyroid adenoma or hyperplasia which predominantly affect women, parathyroid carcinoma appears to be equally distributed between genders [1, 3]. The etiology of parathyroid cancer is unknown. There is a reported increased incidence in cases with previous neck irradiation (3, 9, 10). Of the 358 patients reviewed by Koea and Shaw seven (1,9%) reported an autosomal dominant inheritance pattern of familial hyperparathyroidism [3]. 

The diagnosis of parathyroid carcinoma in the absence of regional or distant metastases is a challenging issue. Parathyroid carcinomas, as opposed to other endocrine tumors that become less hormonally active when malignant, are hyperfunctional and characterized by severe elevations of serum calcium with associated renal and bone symptoms. Symptoms and signs are related with the metabolic consequence of hypercalcaemia, then patients usually present with a severe form of hyperparathyroidism at diagnosis, such as bone disease, renal disease, or hypercalcemic crisis, in contrast to relatively asymptomatic presentation of benign parathyroid disease. Very few cases of nonfunctional parathyroid cancers have been reported in the literature accounting for approximately 1.9% of parathyroid tumors and represent a prognostic indicator of poor outcome [3]. The level of total serum calcium is significantly elevated in all series of parathyroid carcinoma, with the mean values between 14 and 15 mg/dL [1, 2, 7]. Similarly the PTH level in parathyroid carcinoma is consistently higher than for benign parathyroid disease [1, 7]. Up to 14% of patients with parathyroid carcinoma may present with hypercalcemic crisis [11]. Another common feature of parathyroid carcinoma that should alert the clinician is the presence of a palpable neck mass, associated with malignancy in about 35% of patients [6]. The most frequent complaints are fatigue, weakness, anxiety, nausea, vomiting, polyuria, and polydipsia [2]. Renal colic, bone pain, and pathological fractures are also common features of parathyroid cancer [3]. It is important to note the high incidence of concomitant bone and stone disease that occurs in parathyroid cancer, that is very unusual in primary hyperthyroidism. In our series all patients had at least renal or skeletal disease, and half of them had simultaneous symptoms. A comparison between clinical features of parathyroid carcinoma and benign disease is summarized in Table 3. 

Diagnosis is not made easily even on histology. Schantz and Castleman have established the criteria for the histological diagnosis of parathyroid carcinoma. These are the presence of fibrous capsule or fibrous trabeculae, a trabecular or rosette-like cellular architecture, the presence of mitotic figures, and the presence of capsular or vascular invasion [9]. Occasionally a patient with a parathyroid carcinoma may remain undiagnosed until the tumor either recurs locally or develops distant metastasis. 

The intraoperative diagnosis of parathyroid cancer could also be not easy. Whereas parathyroid adenomas tend to be soft, oval, and brownish red to tan in appearance, the macroscopic operative features of parathyroid carcinoma are characterized by lobulated firm mass, surrounded by fibrous grayish-white capsule that adheres tenaciously to thyroid lobe or adjacent cervical tissues (strap muscles, recurrent laryngeal nerve, esophagus, and trachea). Of the 358 patients in the series reported by Koea and Shaw the diagnosis of carcinoma was made or suspected intraoperatively by the surgeon in 178 (49,8%) because of invasion of the surrounding tissues [3]. In 46 of these cases a dense fibrous capsule was also noted. The most common sites of invasion were the ipsilateral thyroid gland 89%, strap muscles 71%, ipsilateral recurrent laryngeal nerve 26%, oesophagus 18%, and trachea 17%. On the contrary one of the largest series of 286 cases reported from the American National Cancer Data Base concluded that a majority of cases had not been recognized at surgery and resections were “incomplete and piecemeal” in 86% of patients [5]. 

The overall survival at 5-year and 10-year was 85% and 49%, respectively, in this series. Further studies report similar 5 years survival rate with higher 10 years survival rate (77%), probably related to improvements in overall general supportive medical care and prevention of fatal hypercalcemia [12]. In most series long term results in terms of local recurrence and distant metastasis are significantly improved when an *en bloc* excision including the ipsilateral thyroid lobe is performed as opposed to cases in which only the parathyroid cancer is removed [1–3, 6, 10, 13]. In particular in a multivariate analysis, Sandelin and coll. showed that patients treated initially with more extensive surgery had a longer survival and a longer relapse-free period than patients treated with tumor resection alone [6]. Similarly the analysis of literature of Koea and Shaw reported an overall 8% evidence of local recurrence after *en bloc* resection compared to a 51% incidence after a standard parathyroidectomy [3]. In this review survival data demonstrated that *en bloc* resection of parathyroid carcinoma at first presentation is associated with better local disease control and significantly improved long-term survival. The most effective treatment of parathyroid carcinoma remain surgical. Failure to eradicate the carcinoma at the first operation often leads to repeated local recurrence or distant metastasis. The initial *en bloc* resection, avoiding rupture of the tumor capsule and spillage of tumor cells, represents the best chance for cure [1]. In the series reported by the authors there was only one patient with relapse of disease. He was the patient that did not receive *en bloc* resection because diagnosis of cancer was not suspected pre- or intraoperatively. Controversy exists as to whether the patient in whom malignancy is recognized after tumorectomy requires reoperation with resection of all structures adjacent to where the tumour was originally located. Some investigators have suggested that in these cases reoperation may be postponed until tumor recurrence is recognized by rising calcium levels [1, 2]. Current literature, however, suggests that conservative resection is associated with a significant risk of capsule rupture and subsequent local dissemination of the tumor, therefore reoperation with ipsilateral total thyroid loboistmectomy is recommended [3]. For the same reason a preoperative aspiration biopsy or an intraoperative incisional biopsy of the mass must be avoided, otherwise local dissemination of tumor cells may occur [14, 15]. 

For this reason the recent practice of minimally invasive parathyroidectomy, which is appropriate in the vast majority of patients with PHPT, should be altered in suspicious situations. 

Some authors reported that despite infiltration macroscopically or adherence of the tumor to adjacent structures is often associated with malignant tumors, it could be sometimes present in benign lesions [16, 17]. In our series of 144 patients an *en bloc* resection of the parathyroid with the ispilateral thyroid lobe and adjacent adherent structures were performed in four cases because of features of tenaciously adherent parathyroid gland. As reported in three of this patients a diagnosis of parathyroid carcinoma or atypical adenoma was confirmed, only in one case the final histological examination revealed the diagnosis of parathyroid adenoma with associated thyroiditis. The data of literature and our experience showed that it is preferable to treat all these patients as parathyroid carcinoma than to miss the opportunity for surgical cure.

Although the reported incidence of cervical lymph node metastasis is <20% the most of authors recommended routine dissection of trachea-esophageal groove, but an extensive lateral neck dissection is indicated only when there is macroscopic spread to the anterior cervical nodes [1, 2, 4].

The patient who succumbed to parathyroid carcinoma typically died from metabolic consequences and not directly from malignant growth. For this reason surgical treatment to debulk parathyroid carcinoma, if possible, is indicated. Before reoperation a vigorous effort to locate the recurrent carcinoma is essential thereafter aggressive re-resection of neck recurrence or metastasis despite is often palliative and may offer improvement of symptoms for a long time [1]. Chemotherapy and radiotherapy have limited role in the palliative management of the patient with unresectable neck disease or metastatic disease [3]. 

In conclusion the preoperative evaluation of a patient with severe hypercalcemia and high PTH levels should include the possible diagnosis of parathyroid carcinoma, especially in symptomatic patients or in case of palpable neck mass. The surgeon will often report that dissection of the mass was surprisingly difficult in the patient with parathyroid carcinoma, with unexpected adherence to the surrounding soft tissues. In case of preoperative high suspicion of parathyroid carcinoma or intraoperative findings of firm adherent mass, an *en bloc* resection of the mass with total ipsilateral thyroid loboistmectomy and central compartment lymphadenectomy should be performed. Indeed attempts to separate the parathyroid tumor in such instances will risk effraction of the capsule and result in tumor spillage and further recurrence of disease and definitive lack of possible cure of the disease.

## Figures and Tables

**Table 1:** Diagnoses in primary hyperthyroidism.

Diagnosis	*n*	%
Adenoma	123	85,41%
Hyperplasia	17	11,80%
Carcinoma	3	2,08%
Atypical adenoma	1	0,69%

**Table 2:** Main clinical features of patients with parathyroid carcinoma (PC) and atypical adenoma (AA).

	Patient 1 (PC)	Patient 2 (PC)	Patient 3 (PC)	Patient 4 (AA)
Age	46	69	64	54
Gender	F	M	M	M
Symptoms	Renal + Bones – Psychiatric +	Renal + Bones + Psychiatric +	Renal + Bones + Psychiatric −	Renal − Bones + Psychiatric +
Palpable mass	No	No	Yes	Yes
Calcium (mg/dL)	14,0	13,0	14,2	13,2
PTH (pg/mL)	898	184	620	475
Type of resection	*En bloc* resection with the ipsilateral lobe + paratracheal lymphadenectomy	(1) MIVAP (2) After 3 weeks thyroid loboistmectomy + paratracheal lymphadenectomy	*En bloc* resection with the ipsilateral lobe	*En bloc* resection with the ipsilateral lobe
Affected gland	Left superior	Left superior	Left inferior	Right inferior
Tumor size	2,7 × 1,4 × 2,5 cm	1,9 × 1,7 × 1,4 cm	3,6 × 2 × 2,7 cm	3 × 1,7 × 1,2 cm
Outcome	Alive, no evidence of disease at 60 months of followup	Alive, surgically treated lung metastasis, rise in calcium/PTH levels without evidence of recurrence at imaging investigations at 36 months of followup	Alive, no evidence of disease at 48 months of followup	Died for other disease

**Table 3:** Typical features of PHPT secondary to parathyroid carcinoma versus parathyroid benign disease.

	Parathyroid carcinoma	Parathyroid benign disease
Average age	48	55
Female/male ratio	1 : 1	3,5 : 1
Tumor size >3 cm	Frequent	Very rare
Serum calcium	14–16 mg/dL	11-12 mg/dL
PTH	Markedly elevated	Mildly elevated
Asymptomatic	<2%	40–80%
Palpable neck mass	38%	<2%
Hoarseness	1–14%	None
Renal and skeletal involvement	30–50%	<5%
Hypercalcemic crisis	14%	<2%
Intraoperative findings	Lobulated firm mass, surrounded by fibrous grayish-white capsule that adheres tenaciously to thyroid lobe or adjacent cervical tissues	Soft, oval, and brownish red

(From ref. [1–3, 6, 9, 10]).
